# History of malaria control in Tajikistan and rapid malaria appraisal in an agro-ecological setting

**DOI:** 10.1186/1475-2875-7-217

**Published:** 2008-10-26

**Authors:** Barbara Matthys, Tohir Sherkanov, Saifudin S Karimov, Zamonidin Khabirov, Till Mostowlansky, Jürg Utzinger, Kaspar Wyss

**Affiliations:** 1 Swiss Centre for International Health, Swiss Tropical Institute, P.O. Box, CH-4002 Basel, Switzerland; 2 Republican Centre of Tropical Diseases, Ministry of Health, Alischera Nawon street 5/4, Dushanbe, Tajikistan; 3 Institute of Zoology and Parasitology, 734025, Box 70, Dushanbe, Tajikistan; 4 Project Sino, Prospect Rudaki, 5-I prozed, dom 1, Dushanbe, Tajikistan; 5 Department of Public Health and Epidemiology, Swiss Tropical Institute, P.O. Box, CH-4002 Basel, Switzerland

## Abstract

**Background:**

Reported malaria cases in rice growing areas in western Tajikistan were at the root of a rapid appraisal of the local malaria situation in a selected agro-ecological setting where only scarce information was available. The rapid appraisal was complemented by a review of the epidemiology and control of malaria in Tajikistan and Central Asia from 1920 until today. Following a resurgence in the 1990s, malaria transmission has been reduced considerably in Tajikistan as a result of concerted efforts by the government and international agencies. The goal for 2015 is transmission interruption, with control interventions and surveillance currently concentrated in the South, where foci of *Plasmodium vivax *and *Plasmodium falciparum *persist.

**Methods:**

The rapid malaria appraisal was carried out in six communities of irrigated rice cultivation during the peak of malaria transmission (August/September 2007) in western Tajikistan. In a cross-sectional survey, blood samples were taken from 363 schoolchildren and examined for *Plasmodium *under a light microscope. A total of 56 farmers were interviewed about agricultural activities and malaria. Potential *Anopheles *breeding sites were characterized using standardized procedures. A literature review on the epidemiology and control of malaria in Tajikistan was conducted.

**Results:**

One case of *P. vivax *was detected among the 363 schoolchildren examined (0.28%). The interviewees reported to protect themselves against mosquito bites and used their own concepts on fever conditions, which do not distinguish between malaria and other diseases. Three potential malaria vectors were identified, i.e. *Anopheles superpictus*, *Anopheles pulcherrimus *and *Anopheles hyrcanus *in 58 of the 73 breeding sites examined (79.5%). Rice paddies, natural creeks and man-made ponds were the most important *Anopheles *habitats.

**Conclusion:**

The presence of malaria vectors and parasite reservoirs, low awareness of, and protection against malaria in the face of population movements and inadequate surveillance may render local communities vulnerable to potential epidemics. To attain malaria transmission interruption in Tajikistan by 2015, there is a need for rigorous surveillance along with strengthening of primary health care facilities for effective case management, and possibly a more differentiated vector control strategy based on additional local evidence.

## Background

### Country background

The Republic of Tajikistan is a land-locked mountainous country in Central Asia with an estimated population of 6.9 million; nearly 75% living in rural areas [1]. Although the economy has grown considerably since 1997, two-thirds of the population live on less than US$ 2.15 per day [2]. The economy strongly depends on cotton, aluminium and hydropower exports [3]. The climate is continental but close to Mediterranean with a dominant winter-spring precipitation, hot and dry summers and cold winters. The ratio of temporary *versus *permanent arable land is approximately 1:5 with cotton being the most important crop. Since the beginning of the 1990s, cotton production has partially been replaced by irrigated rice farming on a small and fluctuating part of the agricultural land [4].

### Malaria epidemiology and control in Tajikistan

**Figure 1:**
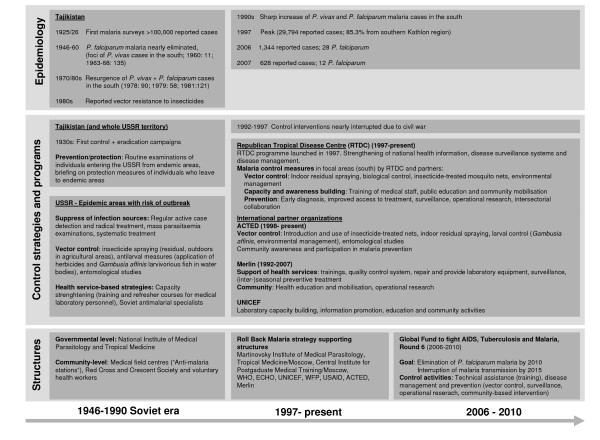
Malaria epidemiology and control strategies from 1920 to present.

The history of malaria and its control can be divided into the Soviet era (1920 to the early 1990s) and the period after the independence of Tajikistan (1991 to date). The epidemiology, control strategies employed, and responsible programs and structures are detailed in Figure 1.

**Figure 2:**
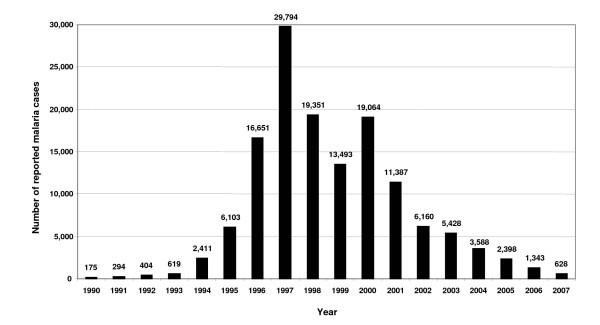
**Reported malaria cases in Tajikistan between 1990 and 2007**. Source: Merlin UK.

Malariological surveys carried out in 1925/26 revealed that inhabitants of valleys were at high risk [5,6]. Only a few isolated foci of *Plasmodium vivax *and *Plasmodium falciparum *persisted in southern parts of the country bordering endemic areas. In the late 1970s and 1980s, the reported number of *P. vivax *and *P. falciparum *cases increased, mainly in the South. A sharp rise was observed from 1994 (2,411 cases) onwards with a peak occurring in 1997 (29,794 cases). Subsequently, the number of cases declined; in 2007 there were 628 reported cases (Figure 2). Although these figures should be interpreted with caution due to the lack of rigorous surveillance, the data indicate a major improvement of malaria in recent years.

The first malaria control campaigns were conducted in the 1930s [6]. Under Soviet rule, preventive measures were implemented across the whole territory. Control strategies in epidemic areas of elevated risk of infection were implemented, including active case detection and treatment, vector control, and health service-based control strategies (Figure 1) [5-10].

Between 1992 and 1997, malaria control efforts in Tajikistan almost came to a halt due to a five-year civil war and a consequent economic ruin, which led to a disruption of health care services including disease control activities [11]. The Republican Tropical Disease Centre Program (RTDC), a department of the State Epidemiological Service (SES), was reactivated by the Tajik Ministry of Health (MoH). The mandate of RTDC is to implement malaria control strategies, to strengthen the national health information and disease surveillance and management systems, to conduct operational research, and to facilitate intersectoral collaboration. Joint activities between the Tajik MoH and the NGO 'Agency for Technical Cooperation and Development' (ACTED) and the British NGO Merlin UK (1992–2007) [6,9,12-17] resulted in a considerable decline of the number of malaria cases in the new millennium [6,12]. Activities have been reinforced since 2006 by substantial funding for malaria control through the Global Fund to Fight AIDS, Tuberculosis and Malaria (GFATM) [17] (Figure 1).

Currently, the South of Tajikistan is considered to be malaria endemic, whereas the Centre and North are prone to epidemics [9,18]. Transmission of *P. vivax *malaria occurs between April and October with a peak in August/September [19]. Transmission of *P. falciparum *malaria occurs from June/July to December with a peak in October. The predominant malaria vectors in Tajikistan and neighbouring countries are *Anopheles superpictus *and *Anopheles pulcherrimus *[20] and, to a lesser extent, *Anopheles hyrcanus*, *Anopheles maculipennis *ss and *Anopheles claviger *[21]. Heavy rainfalls and suitable temperatures in the spring favor the development of first mosquito breeding sites. During the hot and dry summer, irrigated cotton and rice fields in the Western plains are supplied by snow and glacial melting water from the Pamir mountains [22]. A steady increase of the annual average temperature, changes in agricultural land use, in particular the increase of rice cultivation, and decaying irrigation systems provide breeding grounds for mosquitoes [9,11,22-24]. An amplification of malaria vectors prompted the government to almost half the areas of irrigated rice in the entire country between 2000 and 2003.

It has also been suggested that changing malaria distribution patterns observed in recent years were influenced by cross-border and in-country population movements and deteriorating socioeconomic conditions, as a result of the instable political situation [5]. Persistent malaria cases in rice growing communities in Western Tajikistan, which is also known for population dynamics due to its economical prosperity in the recent past, were at the root of the present study. The objectives were to review the history of the epidemiology and control of malaria in Tajikistan and the broader sub-region of Central Asia, and to conduct a rapid appraisal of the malaria situation in a selected agro-ecological setting in western Tajikistan.

## Methods

### The rapid malaria appraisal methodology

The rapid urban malaria appraisal (RUMA) methodology was developed by the Swiss Tropical Institute as a cost-effective tool to assess the malaria situation in urban settings of sub-Saharan Africa. It consists of a literature review; collection of health statistics; risk mapping; school parasitemia surveys; health facility-based surveys; and a summary of the health care system. So far, the RUMA methodology has been evaluated for its feasibility, consistency and usefulness in four urban contexts of sub-Saharan Africa [25-29]. The appraisal reported here was conducted by adaptating the RUMA methodology to an area outside Africa.

### Study area

In August and September 2007, a cross-sectional survey was carried out in rural communities located south of the centre of Tursunzoda district in Western Tajikistan. The district is economically one of the most developed in the country. The largest aluminium smelting plant in Central Asia, which is one of the nation's most important industrial assets, is located there. Agriculture relies primarily on cotton and rice cultivation, and represents the main economic activity in rural areas where 80% of the district's population lives. The number of inhabitants of the six communities ranged between 900 and 2,700 in October 2006, according to an official population database from the Tajik government.

### Collaboration and ethical approval

The study was carried out within the frame of a Tajik-Swiss Health Reform and Family Medicine Project that aims at strengthening family medicine services and health reforms [30,31]. Collaborating partners were the RTDC and the Tajik Institute of Zoology and Parasitology (IZP).

Ethical approval was obtained from the Tajik MoH and, at district level, the study was cleared by the primary health care manager. Written informed consent was received from parents or legal guardians of participating children and by the farmers interviewed.

### Cross-sectional malaria and questionnaire survey

In a first step, the planned activities were discussed with the head of the municipal administration, which aggregates between 10 and 20 villages. Six communities were selected based on the presence of primary schools, and of rice paddies in close proximity to the inhabited area. School directors and village authorities were invited to discuss the surveys and to inform their communities.

During the school-based survey, 60 schoolchildren aged 7–10 years were examined for malaria parasitemia. A laboratory technician took a finger prick blood sample and prepared a thick and thin blood film on a microscope slide. The blood slides were air-dried and transferred to the RTDC laboratory in Dushanbe. Slides were stained with Giemsa following routine procedures and read under a light microscope using 100× magnification by experienced laboratory technicians in order to identify *Plasmodium *at species level and to quantify parasitemia, by counting the number of parasites per 200 white blood cells (WBC) in a thick film (assuming 8,000 WBC per μL of blood). A random sample of 73 slides was cross-checked by a senior laboratory technician at the Centre Suisse de Recherches Scientifiques (Abidjan, Côte d'Ivoire) for quality control and judged to be acceptable.

Additionally, each child was interviewed about mosquito bites, mosquito net use and water sources and water storage in their household, and the axillary temperature was measured. A questionnaire was administered to the school directors of each community to assess the sociodemographic and socioeconomic situation.

### Mosquito breeding site characterization and farmers' land use

Between 10 and 12 accessible breeding sites of potential malaria vectors in rice paddies and other agricultural land were mapped within a range of 1–2 km of each village by two entomologists using a hand-held global positioning systems (GPS) receiver (eTrex, Garmin International Inc.; Olathe, USA). Habitat characteristics (i.e. crop type, water movement, turbidity, sunlight, presence of vegetation and potential predators) and the phenological development stages of rice plants were recorded [32]. At each site, 10 dips were taken using a 500 ml measuring cup (dipper). "Presence" of *Anopheles *larvae was defined by the identification of at least one *Anopheles *larvae in any of the 10 dips. The same sampling methods were used to assess *Culex *larvae, pupae of *Anopheles *and *Culex*, and for potential predators. The presence of potential mosquito larval predators *Gambusia affinis *and aquatic snails was also noted. Third and fourth instar larvae and pupae were collected to obtain hatched adult mosquitoes, and identified morphologically under a light microscope by a senior entomologist from IZP.

Two interviewers identified eight to ten farmers during their work in the field and asked them about agricultural land use and activities, overnight stays in the field, perception of the current local malaria situation and personal protection measures against malaria. In addition, staff from medical centres was interviewed about reported malaria cases and treatment in their facility.

Questionnaires and the breeding site appraisal form had been utilized in previous school and farming-based malaria surveys in urban settings of Africa [25,33-35]. The tools were adapted to the current epidemiological setting and pre-tested in one of the communities before use.

### Data management and analysis

Data were entered and validated using version 3.1 of the EpiData Software (EpiData Association; Odense, Denmark). Statistical analysis was performed with version 9.0 of the STATA software package (STATA Corporation; College Station, TX, USA). Images of the study area were downloaded from Google Earth Software (Boston, MA, USA), geo-referenced in version 9.0 of Arc Map™ software (Environmental Systems Research Institute, Redlands; CA, USA) and utilized for the display of potential *Anopheles *breeding sites.

## Results

### Primary health care services

There were two rural health centres and two health houses operating at the time of the survey. Between May and September 2007, 38 laboratory-confirmed cases of *P. vivax *malaria were reported in the district of Tursunzoda. Not a single case of *P. falciparum *was reported in 2006 and 2007.

### School parasitemia survey

The findings from the school-based parasitemia survey are summarized in Table 1. Overall, 366 schoolchildren participated, and complete malariological data were obtained from 363 children. The mean age was 7.9 years (SD = 0.8 years). The majority of the children had an axillary temperature < 37.0°C, whereas nine children had an axillary temperature above 37.5°C. One case of *P. vivax *was detected in an 8-year-old boy (parasitemia = 3,200 parasites per μL of blood), resulting in a prevalence of 0.28%. Less than 10% of the children declared having slept under a mosquito net the previous night. At village level, the reported use of mosquito nets varied between 1.7% and 18.3%.

**Table 1:** Major characteristics of the school-based parasitemia surveys from 6 communities in western Tajikistan, September 2007

**Parameter**	**Nr. (%)**
	
Total children	366 (100)
Sex	
Boy	179 (48.9)
Girl	187 (51.1)
Age (years)	
6–7	125 (34.2)
8	182 (49.7)
9–10	59 (16.1)
Axillary temperature °C	
< 37.0	262 (71.6)
37.0–37.4	95 (26.0)
> 37.4	9 (2.5)
Presence *Plasmodium vivax*	
No	361 (98.9)
*P. vivax*	1 (0.3)
No examination	3 (0.8)
	
Slept under a mosquito net last night	30 (8.2)
Not known	2 (0.6)
Source of household water	
Water tap	60 (16.4)
Cistern	46 (12.6)
Public well	25 (6.8)
Irrigation canal	217 (59.3)
River, creek	5 (1.4)
Not known	13 (3.6)
Source of drinking water	
Water tap	134 (36.6)
Cistern	182 (49.7)
Private well	49 (13.4)
Irrigation canal	1 (0.3)
Storage of household water	
Pail, churn	364 (99.4)
Not known	2 (0.6)
Open water storage container	59 (16.1)
Not known	7 (1.9)

The main sources of drinking water were cisterns, water taps and public wells. Sources of household water were mainly irrigation canals at the border of agricultural fields or in the village to irrigate vegetable gardens and fruit trees within yards. Household water was also drawn from cisterns, public wells and from nearby rivers. Household and drinking water was stored in pails, cans, barrels and water tanks. Approximately one-sixth of the containers were reported not to be covered.

### Mosquito breeding sites assessment

A total of 73 potential mosquito breeding sites within a range of 1–2 km of the villages were characterized (Table 2). Habitat types were "rice paddy" (n = 50), "natural creek" (n = 9), "artificial hole" (n = 7) and "irrigation canal" (n = 7). The majority of the examined sites were classified as large (perimeter > 10 m) with transparent water with about one-third of the sites shaded. Vegetation, mainly herbs and grass, was present in over 60% of the sites. Potential larval predators found were *Gambusia *spp, backswimmers, dragonfly larvae and water snails. *Anopheles *larvae were detected in 58 sites (79.5%), *Culex *larvae in 39 (53.4%), and *Aedes *larvae in three (4.1%). Pupae were found in 12 sites (16.4%).

**Table 2:** Investigated mosquito breeding sites (n = 73) in rice-growing communities, western Tajikistan, September 2007

**Parameter**	**Number (%)**
	
**Total**	73 (100.0)
**Habitat type**	
Rice paddy	48 (65.8)
Phenological stage "panicle differentiation – pollinisation"	13 (28.3)
Phenological stage "panicle extruding and maturation"	32 (69.6)
Phenological stage "idle plot"	1 (2.2)
	
Creek, source	9 (12.3)
Irrigation canal/drain	8 (11.0
Man-made pond (watering place)	8 (11.0)
	
**Physical parameters**	
**Perimeter**	
< 1 m	11 (15.1)
1–10 m	3 (4.1)
> 10 m	59 (80.8)
**Water movement**	
Stagnant	71 (97.3)
Flowing	2 (2.7)
**Turbidity**	
Transparent^a^	56 (76.7)
Turbid^b^	17 (23.3)
**Sunlight**	
Shade: 1–10%	34 (46.6)
Shade: 11–50%	14 (19.2)
Shade: > 50%	25 (34.3)
	
**Biological parameters**	
Presence of vegetation	44 (60.3)
Herbs/grass	32 (43.8)
Algae	26 (35.6)
Floating vegetation	9 (12.3)
	
**Potential predators**	
All predators	45 (61.6)
Backswimmers/water insects	21 (28.8)
Dragonfly larvae	21 (28.8)
Water snails	12 (16.4)
Larvivorious fish *Gambusia *spp	7 (9.6)

^a^Agricultural trench, fosse and puddle between vegetable patches

^b ^Bottom of dipper still visible

^c ^Bottom of dipper not visible

**Figure 3:**
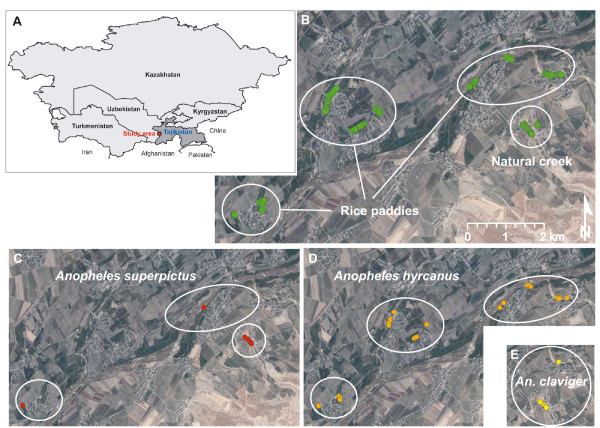
**Study area and examined potential *Anopheles *breeding sites in western Tajikistan, September 2007**. A Study area. B Examined potential *Anopheles *breeding sites. C Identified *Anopheles superpictus*. D Identified *Anopheles hyrcanus*. E Identified *Anopheles claviger*.

Rice paddies and creeks were frequently inhabited by *Anopheles *larvae and pupae (Table 3). Larvae from 36 samples were determined at species level. Adult mosquitoes of *An. hyrcanus *and *An. superpictus *were identified from six, and *An. claviger *from five samples. Figures 3A and 3B show the study area and the breeding sites examined. *An. hyrcanus *was mainly found in rice paddies (Figure 3D), whereas *An. superpictus *and *An. claviger *cohabited in a natural creek located in a valley with cattle and horse pastures along its borders (Figure 3C and 3E).

**Table 3:** Identified larvae and pupae of *Anopheles *and *Culex *in rice-growing communities, western Tajikistan, September 2007

**Habitat type**	**Total sites**	***Anopheles *larvae**	***Culex *larvae**	***Aedes *larvae**	**Pupae**^a^
					
Rice paddy	48	39 (81.3)	18 (37.5)	0	6 (12.5)
Natural creek	9	9 (100.0)	9 (100.0)	3 (33.3)	5 (55.6)
Irrigation canal/drain	8	4 (50.0)	4 (50.0)	0	0
Man-made pond	8	6 (75.0)	8 (100.0)	0	1 (12.5)
					
Total	73	58 (79.5)	39 (53.4)	3 (4.1)	12 (16.4)

^a^*Anopheles *and *Culex*

### Interviews with farmers

Overall, 56 farmers were interviewed on agricultural activities and malaria. All farmers were engaged in farming for at least two years and up to 54 years. Irrigated agriculture has been practiced in this region for centuries according to the interviewees, and rice cultivation partially determined economic wealth. Rice cultivation included one crop cycle of three to five months from May to September and was grown along with vegetable-based and rain-fed crops between April and November. Thirteen interviewed farmers reported periodical overnight stays outdoors during the main cultivation season, particularly within the harvest period from June to September. Mainly adults stayed in the fields overnight, but two farmers reported that their entire families were involved. One family permanently moved to the fields during the harvest period. Reasons were irrigation, competition for irrigation water, harvesting and the protection of crops against theft and damage by wild animals.

Malaria was perceived to be less prevalent in 2007 than in previous years by the majority of the interviewees. Nevertheless, every third person reported "malaria" having occurred in their family or neighborhood within the last two months. "*Tif*" (typhoid fever) was pointed out as an increasing and serious health problem. However, the respondents did not distinguish between "*malaria*" and "*tif*". The unambiguous Russian term "*maliaria*" was almost unknown, but the terms "*tablarza*" (Tajik), and "*bezgak*" (Uzbek) were mentioned, both meaning "shivering". The two latter terms related to a disease causing high fever. The majority of the respondents stated the presence of mosquitoes and three-quarter of the children reported having had mosquito bites within the last two days. Measures to protect against mosquito bites were burning cow dung and a herb locally named "*Ispand*" (*Peganum harmala*), obtained from local markets or collected, to smoke out mosquitoes. Antiseptic and insect repellent effects have repeatedly been attributed to the herb "*Ispand*", which was qualified as a "medicinal plant" [36]. *"Sleeping under a mosquito net last night*" was reported by only seven interviewees.

## Discussion

### Findings from and shortcomings of the rapid malaria appraisal

The malaria situation was assessed in a selected agro-ecological setting in western Tajikistan during the peak of malaria transmission. A rapid appraisal methodology was employed in six communities of irrigated rice cultivation, using a cross-sectional school-based parasitemia survey, supplemented by interviews with farmers on agricultural activities and malaria and characterization of *Anopheles *breeding sites.

The present study is an extension of the RUMA methodology initially developed and validated in African urban settings [26], and further adapted to fit an Asian context. At present, the findings are only representative for this specific agro-ecological setting of Tajikistan, partially explained by selecting study villages in close proximity to irrigated rice paddies, rather than adopting a random sampling strategy. Moreover, the sample size was relatively small and the study of short duration. A limitation of the rapid appraisal methodology was related to the school-based malaria parasitemia survey. Malariological surveys in school settings have been conducted mainly in highly endemic areas. Studies from low prevalence settings are scarce because of required large sample sizes or longitudinal study designs [37]. A more reliable assessment might be obtained by monitoring malaria incidence through repeated surveys or active case detection in schools including absent children, complemented by passive case detection in health centres during the peak transmission period [38,39]. The present methodology might further be developed and standardized in order to serve as a "rapid malaria appraisal" tool to assess the malaria situation in local epidemic settings where pre-existing information is scarce.

The existence of malaria was confirmed by the detection of one *P. vivax *case among 363 children examined. This finding does, however, not prove local transmission, as infection could have taken place elsewhere, e.g. in an endemic part in the South of Tajikistan. In view of the low positivity rate (0.28%), it is likely that there is little acquired immunity against malaria in the area. Nevertheless, it has been suggested that asymptomatic *P. vivax *and *P. falciparum *carriers in Tajikistan might account for up to 90% of all malaria cases and it was estimated that the country harboured over a hundred thousand symptomatic plus asymptomatic infections in 2005 [11]. The number of malaria cases in official malaria statistics from local health facilities in the study area might be under- or over-reported. In fact, some resident sources suggested that health services are under pressure from communities to keep the case number statistics low, since rice growing was prohibited by the regional government within a buffer zone of several km around inhabited areas in 2002. The presence of foci of malaria transmission in the region were suspected by the same sources. In addition, fever patients do not necessarily refer to health care services, and patients are not routinely examined for malaria because microscopic diagnosis is time consuming and blood films can only be examined in district laboratories. Even if blood samples are taken at community health centres, patients are often referred directly to the next district health centre. Official MoH guidelines for case management and prevention have been developed for health services at district and community level (Order of the MoH Nr 192 from 22 April 2004). Laboratory-confirmed diagnosis is recommended for patients with a recent history of fever (within three days) or recurrent fever irrespective of treatment.

*An. superpictus*, *An. hyrcanus *and *An. claviger *were detected in rice paddies, a natural creek and in man-made watering tanks during the two-week field work. All three *Anopheles *species have been reported as potential vectors of *P. vivax *malaria, to be widespread in Central Asia, and to have preferences for rice paddies as breeding habitats [21,40,41]. *An. superpictus *and *An. hyrcanus *may transmit *P. falciparum *[41,42]. *An. pulcherrimus*, another important malaria vector occurring in southern Tajikistan, was not found during the study. This species was observed in water tanks and irrigated fields in Pakistan [43] and in rice paddies, pools and marshes in Iran [44].

The interviewees complained about mosquitoes in their house yards and concomitant nuisances and explained how they protected themselves against mosquito bites (e.g. burning of herbs and cow dung to smoke out mosquitoes). However, farmers did not distinguish between "tif", "malaria" and fever, emphasizing the importance of enhancing the understanding of local perceptions and concepts on malaria and other diseases, and on local health-seeking behavior [45].

Although this study could not confirm local malaria transmission, it seems likely, considering the entire evidence, that there is a risk of malaria transmission related to mosquito breeding of *An. superpictus*, *An. hyrcanus *and *An. claviger *in irrigated fields and open water sources in household yards and villages and low awareness of malaria and prevention measures. Overnight stays outdoors has been shown as a risk factor for malaria in South Asia [46,47] South America [48] and Africa [33,49], and it remains to be investigated, whether it is a risk factor in this setting. Other potential risk factors contributing to a re-introduction of malaria are human mobility, as reported from Pakistan and India [50-52]. Booming house construction observed in some of the villages during the field work was, according to explanations from local people, related to systematic "re-settlement" of populations from other regions of Tajikistan.

### Malaria elimination in the Central Asian Region in the near future?

The term "elimination" is defined as "cessation of the transmission of a disease in a single country or continent" whereas "eradication" refers to "a reduction of the worldwide incidence to zero as a result of deliberate efforts" (International Task Force for Disease Eradication 1989–1992) [11]. In 2005, the Tashkent Declaration ("The move from malaria control to elimination 2006–2015") was endorsed by all countries of the WHO European Region, including Central Asia where malaria is still occurring [11]. The overall goal of the regional strategy in Central Asia is to interrupt the transmission of malaria by 2015. Specific objectives comprise incidence and prevalence reduction, *P. falciparum *elimination, prevention of epidemics and maintenance of malaria-free regions. National health services' capacity and capability are being strengthened and community actions mobilized. For Tajikistan, the rationale is supported by the observed reduction of the number of malaria cases between 1999 and 2006 and the reported absence of autochthonous *P. falciparum *cases during the eradication era, attributed to control interventions, political will, and application of efficacious tools (e.g. combined pyrethroid-based insecticide spraying and prompt treatment of malaria) [17]. Control strategies in Tajikistan are adapted to national eco-zones: in the Centre and South, malaria mortality is to be prevented, *P. falciparum *transmission interrupted, and active foci of *P. vivax *transmission eliminated [9,53]. However, caution is advisable when interpreting the continuous decline in the number of malaria cases in Tajikistan since 2000 (Figure 2), as for example changes in surveillance practices could greatly influence the number of detected cases.

## Conclusion

With regard to the WHO recommendations on residual malaria transmission foci elimination [54], this rapid malaria appraisal provides some evidence related to possible local malaria transmission in rice growing communities in western Tajikistan. The findings indicate that several of the most important malaria vectors in Central Asia are present and at least to some extent associated with human activity including agricultural land use modifications. It is therefore possible that a parasite reservoirs in humans, autochthonous or imported, could maintain a low level transmission or lead to epidemics. Overnight stays outdoors near agricultural fields is a potential risk factor that should be assessed by further epidemiological and entomological studies.

According to historical experience, elimination might be achieved, provided political will to control malaria continues and financial resources will remain adequate, health services and surveillance are strengthened, and a better understanding of risk factors and transmission is obtained through applied field research. In the face of promising short-term achievements over the last years, malaria elimination in Central Asia also needs a stronger cross-border collaboration [53,55,56]. In western Tajikistan, one essential need is to strengthen primary health care facilities to provide prompt malaria diagnosis on the spot and effective treatment. Transmission in this climate zone is usually highly amenable to indoor residual spraying, if it is carried out properly, with correct timing, and the vectors are susceptible to the insecticide used. Given the potential important role of migrants and of overnight stays near fields, there is a need also for assessing the potential role of preventive measures such as the use of long-lasting insecticide-treated mosquito nets, and community-based environmental control management.

## Competing interests

The authors declare that they have no competing interests.

## Authors' contributions

The study design was conceived and further developed by BM, JU and KW. BM conducted the field work, interpreted the data and drafted the manuscript. JU and KW revised the manuscript. KS enabled the planned activities and TS supervised the field work. ZK and TM contributed to the development of methodological aspects and coordinated parts of the surveys.

## References

[B1] World Urbanization Prospects: The 2007 revision population database. http://esa.un.org/unup/.

[B2] The World Bank: Tajikistan country brief. http://web.worldbank.org/WBSITE/EXTERNAL/COUNTRIES/ECAEXT/TAJIKISTANEXTN/0,,contentMDK:20630697~menuPK:287255~pagePK:141137~piPK:141127~theSitePK:258744,00.html.

[B3] CIA The world factbook: Tajikistan. https://www.cia.gov/library/publications/the-world-factbook/geos/ti.html.

[B4] Gintzburger G, Le Houerou HN, Toderich KN (2005). The steppes of Middle Asia: Post-1991 agricultural and rangeland adjustment. Arid Land Res Manag.

[B5] Pitt S, Pearcy BE, Stevens RH, Sharipov A, Satarov K, Banatvala N (1998). War in Tajikistan and re-emergence of *Plasmodium falciparum*. Lancet.

[B6] Aliev S, Saparova N (2001). Current malaria situation and its control in Tadjikistan. Med Parazitol (Mosk).

[B7] World Health Organization (1956). Methods of malaria control in the USSR.

[B8] Sergiev EG (1960). Progress of malaria eradiction in the USSR in 1959 and targets for 1960. Preparatory data. European conference on malaria eradiction: 1960; Palermo.

[B9] Ejov M (2005). Scaling up the response to malaria in the Europan region. Progress towards curbing an epidemic 2000–2004.

[B10] Zahar AR (1990). Vector bionomics in the epidemiology of malaria. Part II: The WHO European region and the WHO Eastern Mediterranean region. Volume II: Applied field studies. Section I: An overview of the recent malaria situation and current problems. Section II: Vector distribution.

[B11] World Health Organization (2005). Inception meeting on the malaria elimination initiative in the WHO European region.

[B12] Aliev SP (2000). Malaria in the Republic of Tajikistan. Med Parazitol (Mosk).

[B13] Sabatinelli G (2000). The malaria situation in the WHO European region. Med Parazitol (Mosk).

[B14] Sabatinelli G, Ejov M, Joergensen P (2001). Malaria in the WHO European region (1971–1999). Euro Surveill.

[B15] World Health Organization (2000). Second interregional malaria coordination meeting.

[B16] World Health Organization (2001). Progress with roll back malaria in the WHO European region.

[B17] World Health Organization (2006). Cross-border meeting for Central Asian countries and Afghanistan. Elimination of *Plasmodium falciparum *malaria in Central Asia.

[B18] World Health Organization (2006). Malaria strata in Central Asia and Kazakhstan.

[B19] Karimov SS, Kadamov DS, Murodova N (2008). The current malaria situation in Tadjikistan. Med Parazitol (Mosk).

[B20] Rowland M, Mohammed N, Rehman H, Hewitt S, Mendis C, Ahmad M, Kamal M, Wirtz R (2002). Anopheline vectors and malaria transmission in eastern Afghanistan. Trans R Soc Trop Med Hyg.

[B21] Gordeev MI, Ezhov MN, Zvantsov AB, Goriacheva, Shaikevich EV, Karimov SS, Kadamov DS (2004). Supplement to the list of *Anopheles *(*Diptera*, *Culicidae*) mosquitoes of Tadjikistan and the predominant types of vectors in the current foci of malaria in the republic. Med Parazitol (Mosk).

[B22] Rebholz CE, Michel AJ, Maselli DA, Saipphudin K, Wyss K (2006). Frequency of malaria and glucose-6-phosphate dehydrogenase deficiency in Tajikistan. Malar J.

[B23] Lioubimtseva E, Cole R, Adams JM, Kapustin G (2005). Impacts of climate and land-cover changes in arid lands of Central Asia. J Arid Environ.

[B24] Keiser J, De Castro MC, Maltese MF, Bos R, Tanner M, Singer BH, Utzinger J (2005). Effect of irrigation and large dams on the burden of malaria on a global and regional scale. Am J Trop Med Hyg.

[B25] Wang SJ, Lengeler C, Smith TA, Vounatsou P, Diadie DA, Pritroipa X, Convelbo N, Kientga M, Tanner M (2005). Rapid urban malaria appraisal (RUMA) I: epidemiology of urban malaria in Ouagadougou. Malar J.

[B26] Wang SJ, Lengeler C, Smith TA, Vounatsou P, Cisse G, Diallo DA, Akogbeto M, Mtasiwa D, Teklehaimanot A, Tanner M (2005). Rapid urban malaria appraisal (RUMA) in sub-Saharan Africa. Malar J.

[B27] Wang SJ, Lengeler C, Mtasiwa D, Mshana T, Manane L, Maro G, Tanner M (2006). Rapid Urban Malaria Appraisal (RUMA) II: epidemiology of urban malaria in Dar es Salaam (Tanzania). Malar J.

[B28] Wang SJ, Lengeler C, Smith TA, Vounatsou P, Akogbeto M, Tanner M (2006). Rapid Urban Malaria Appraisal (RUMA) IV: epidemiology of urban malaria in Cotonou (Benin). Malar J.

[B29] Wang SJ, Lengeler C, Smith TA, Vounatsou P, Cisse G, Tanner M (2006). Rapid Urban Malaria Appraisal (RUMA) III: epidemiology of urban malaria in the municipality of Yopougon (Abidjan). Malar J.

[B30] Wyss K, Djamalova M (2003). Sharing responsibility for better health care. Carrying forward health sector reform in Tajikistan. MMS Bulletin.

[B31] Tediosi F, Aye R, Ibodova S, Thompson R, Wyss K (2008). Access to medicines and out of pocket payments for primary care: evidence from family medicine users in rural Tajikistan. BMC Health Serv Res.

[B32] Moldenhauer K, Slaton N, Slaton N, Ford L, Bernhardt J, Cartwright R, Gardisser D, Gibbons J, Huitink G, Koen B, Lee F, Miller D, et al (2004). Rice growth and development. Rice Production Handbook.

[B33] Matthys B, Vounatsou P, Raso G, Tschannen AB, Becket EG, Gosoniu L, Cisse G, Tanner M, N'Goran EK, Utzinger J (2006). Urban farming and malaria risk factors in a medium-sized town in Côte D'Ivoire. Am J Trop Med Hyg.

[B34] Matthys B, N'Goran EK, Koné M, Koudou BG, Vounatsou P, Cissé G, Tschannen AB, Tanner M, Utzinger J (2006). Urban agricultural land use and characterization of mosquito larval habitats in a medium-sized town of Côte d'Ivoire. J Vector Ecol.

[B35] Sattler MA, Mtasiwa D, Kiama M, Premji Z, Tanner M, Killeen GF, Lengeler C (2005). Habitat characterization and spatial distribution of *Anopheles sp. *mosquito larvae in Dar es Salaam (Tanzania) during an extended dry period. Malar J.

[B36] Rehmat S, Shah U, Hassan G, Rehman A, Imtiaz A (2006). Ethnobotanical studies of the flora of district Musakhel and Barkhan in Balochistan, Pakistan. Pak J Weed Sci Res.

[B37] Ernst KC, Adoka SO, Kowuor DO, Wilson ML, John CC (2006). Malaria hotspot areas in a highland Kenya site are consistent in epidemic and non-epidemic years and are associated with ecological factors. Malar J.

[B38] Brooker S, Clarke S, Njagi JK, Polack S, Mugo B, Estambale B, Muchiri E, Magnussen P, Cox J (2004). Spatial clustering of malaria and associated risk factors during an epidemic in a highland area of western Kenya. Trop Med Int Health.

[B39] Clarke SE, Brooker S, Njagi JK, Njau E, Estambale B, Muchiri E, Magnussen P (2004). Malaria morbidity among school children living in two areas of contrasting transmission in western Kenya. Am J Trop Med Hyg.

[B40] Severini C, Menegon M, Di Luca M, Abdullaev I, Majori G, Razakov SA, Gradoni L (2004). Risk of *Plasmodium vivax *malaria reintroduction in Uzbekistan: genetic characterization of parasites and status of potential malaria vectors in the Surkhandarya region. Trans R Soc Trop Med Hyg.

[B41] Zahar AR (1990). Vector bionomics in the epidemiology of malaria. Part II: The WHO European region and the WHO Eastern Mediterranean region. Volume II: Applied field studies. Section III: Vector bionomics, malaria epidemiology and control by geographical areas.

[B42] Brooker S, Leslie T, Kolaczinski K, Mohsen E, Mehboob N, Saleheen S, Khudonazarov J, Freeman T, Clements A, Rowland M, Kolaczinski J (2006). Spatial epidemiology of *Plasmodium vivax*, Afghanistan. Emerg Infect Dis.

[B43] Herrel N, Amerasinghe FP, Ensink J, Mukhtar M, Hoek W van der, Konradsen F (2001). Breeding of *Anopheles *mosquitoes in irrigated areas of South Punjab, Pakistan. Med Vet Entomol.

[B44] Djadid ND, Sanati MH, Zare M, Hassanzeho A (2003). rDNA-ITS2 Identification of *Anopheles pulcherrimus *(Diptera: *Culicidae*): Genetic differences and phylogenetic relation with other iranian vectors and its implications for malaria control. Iran Biomed J.

[B45] Beiersmann C, Sanou A, Wladarsch E, De Allegri M, Kouyate B, Müller O (2007). Malaria in rural Burkina Faso: local illness concepts, patterns of traditional treatment and influence on health-seeking behaviour. Malar J.

[B46] Somboon P, Aramrattana A, Lines J, Webber R (1998). Entomological and epidemiological investigations of malaria transmission in relation to population movements in forest areas of north-west Thailand. Southeast Asian J Trop Med Public Health.

[B47] Seng CM, Matusop A, Sen FK (1999). Differences in *Anopheles *composition and malaria transmission in the village settlements and cultivated farming zone in Sarawak, Malaysia. Southeast Asian J Trop Med Public Health.

[B48] Sevilla-Casas E (1993). Human mobility and malaria risk in the Naya river basin of Colombia. Soc Sci Med.

[B49] Hetzel MW, Alba S, Fankhauser M, Mayumana I, Lengeler C, Obrist B, Nathan R, Makemba AM, Mshana C, Schulze A, Mshinda H (2008). Malaria risk and access to prevention and treatment in the paddies of the Kilombero Valley, Tanzania. Malar J.

[B50] Kazmi JH, Pandit K (2001). Disease and dislocation: the impact of refugee movements on the geography of malaria in NWFP Pakistan. Soc Sci Med.

[B51] Rowland M, Rab MA, Freeman T, Durrani N, Rehman N (2002). Afghan refugees and the temporal and spatial distribution of malaria in Pakistan. Soc Sci Med.

[B52] Joshi V, Adha S, Singh H, Singhi M, Dam PK (2006). Introduction, transmission and aggravation of malaria in desert ecosystem of Rajasthan, India. J Vector Borne Dis.

[B53] World Health Organization (2006). Regional strategy: From malaria control to elimination in the WHO European region 2006–2015.

[B54] World Health Organization (2007). Guidelines for the elimination of residual foci of malaria transmission.

[B55] WHO Regional Office for Europe (2008). WHO meeting on progress achieved with malaria elimination in the WHO European region.

[B56] Anonymous (2007). Is malaria eradication possible?. Lancet.

